# Direct expression of active human tissue inhibitors of metalloproteinases by periplasmic secretion in *Escherichia coli*

**DOI:** 10.1186/s12934-017-0686-9

**Published:** 2017-04-28

**Authors:** Ki Baek Lee, Dong Hyun Nam, Jacob A. M. Nuhn, Juan Wang, Ian C. Schneider, Xin Ge

**Affiliations:** 1 Department of Chemical and Environmental Engineering, University of California, Riverside, 900 University Ave, Riverside, CA 92521 USA; 2 Department of Chemical and Biological Engineering, Iowa State University, 3053 Sweeney, Ames, IA 50011 USA; 3 Genetics Development, and Cell Biology Department, Iowa State University, 1210 Molecular Biology, Ames, IA 50011 USA

**Keywords:** Tissue inhibitor of metalloproteinase, Matrix metalloproteinase, Periplasmic expression, Disulfide isomerase, Contact guidance

## Abstract

**Background:**

As regulators of multifunctional metalloproteinases including MMP, ADAM and ADAMTS families, tissue inhibitors of metalloproteinases (TIMPs) play a pivotal role in extracellular matrix remodeling, which is involved in a wide variety of physiological processes. Since abnormal metalloproteinase activities are related to numerous diseases such as arthritis, cancer, atherosclerosis, and neurological disorders, TIMPs and their engineered mutants hold therapeutic potential and thus have been extensively studied. Traditional productions of functional TIMPs and their N-terminal inhibitory domains (N-TIMPs) rely on costly and time-consuming insect and mammalian cell systems, or tedious and inefficient refolding from denatured inclusion bodies. The later process is also associated with heterogeneous products and batch-to-batch variation.

**Results:**

In this study, we developed a simple approach to directly produce high yields of active TIMPs in the periplasmic space of *Escherichia coli* without refolding. Facilitated by disulfide isomerase (DsbC) co-expression in protease-deficient strain BL21 (DE3), N-TIMP-1/-2 and TIMP-2 which contain multiple disulfide bonds were produced without unwanted truncations. 0.2–1.4 mg purified monomeric TIMPs were typically yielded per liter of culture media. Periplasmically produced TIMPs exhibited expected inhibition potencies towards MMP-1/2/7/14, and were functional in competitive ELISA to elucidate the binding epitopes of MMP specific antibodies. In addition, prepared N-TIMPs were fully active in a cellular context, i.e. regulating cancer cell morphology and migration in 2D and 3D bioassays.

**Conclusion:**

Periplasmic expression in *E. coli* is an excellent strategy to recombinantly produce active TIMPs and N-TIMPs.

**Electronic supplementary material:**

The online version of this article (doi:10.1186/s12934-017-0686-9) contains supplementary material, which is available to authorized users.

## Background

Remodeling of the extracellular matrix (ECM) is a critical event in numerous physiological processes (e.g. growth, wound repair, and embryogenesis) [1], and pathological procedures such as arthritis, fibrosis, tumor metastasis, and neurological disorders [2, 3]. Changes in ECM composition are mediated by families of multi-domain extracellular proteinases including MMPs (matrix metalloproteinases), ADAMs (a disintegrin and metalloproteinases), and ADAMTS (a disintegrin and metalloproteinase with thrombospondin motifs). The enzymatic activity and zymogen activation of these metalloproteinases are governed by a group of endogenous proteins named tissue inhibitors of metalloproteinases (TIMPs) [4]. Four human TIMPs have been identified (TIMP-1 to -4, sharing 40% sequence identity), among which TIMP-1/-3 are glycoproteins, whereas TIMP-2/-4 are not glycosylated [5]. All mammalian TIMPs have 12 conserved cysteine residues forming six disulfide bonds that are essential for their functions and structural integrity [1]. N-terminal domains of TIMPs (~125 aa, N-TIMPs) and their C-terminal domains (~65 aa, C-TIMPs) have three disulfide bonds each. N-TIMPs alone fold to stable and native structures carrying full inhibition activities to MMPs and other disintegrin-metalloproteinases [6–8]. C-TIMPs are more divergent, and able to improve inhibition selectivities and binding efficiencies [9]. Investigations with isolated MMP domains and their crystal structures have indicated that N-TIMPs interact directly with the catalytic domain of MMPs to form a stable inactive complex [10].

TIMPs inhibit MMPs with various inhibition constants (*K*_*I*_s) ranging from low to subnanomolar, implicating that they have distinct functions in vivo [1]. In addition to MMPs, several members of the ADAM and ADAMTS families are regulated by TIMPs as well. Among TIMPs, TIMP-3 exhibits the broadest inhibition spectrum. To improve their inhibition potency and selectivity among individual MMPs, N-TIMPs have been engineered by directed evolution using phage display [11] and TIMP chimeras have been designed to expand specificity [12]. As a consequence of their metalloproteinase inhibition activity, TIMPs exhibit biological functions such as promoting cell proliferation, inhibiting angiogenesis, inhibiting transendothelial migration and regulating migration [13–18]. Because of these important physiological processes TIMPs regulate in vivo and in vitro, TIMPs have been the focus of many biochemistry and cell biology researches, and thus steady supplies of active human TIMPs at milligram scales are vital.

Mammalian and insect cells have been employed for producing properly folded recombinant TIMPs [6, 7, 9, 19–21]. With merits of high yield and low cost, bacterial expression systems have also been attempted [21–23]. However, overexpression of (N-)TIMPs in *Escherichia coli*, even when fused with highly soluble MBP (maltose-binding protein), led to improper or incomplete folding or the formation of insoluble inclusion bodies. To obtain their native conformation, the insoluble TIMPs require refolding via manifold denaturation–renaturation steps, i.e. urea solubilization and gradient dialysis. This labor-substantial and time-consuming preparation of TIMPs (usually 3–5 days) significantly impedes studies of TIMPs, especially when TIMP variants are needed [24, 25]. Although successful refolding of TIMPs has been reported in literature [8, 21, 22, 24–29], due to the various chemical–physical properties and characteristics of TIMPs, the optimal procedures must be tailored for each TIMP. In addition to deficient yields, other inevitable limitations often include poor reproducibility, heterogeneous oligomeric products, and difficulty of scale-up [22, 26].

**Fig. 1:**
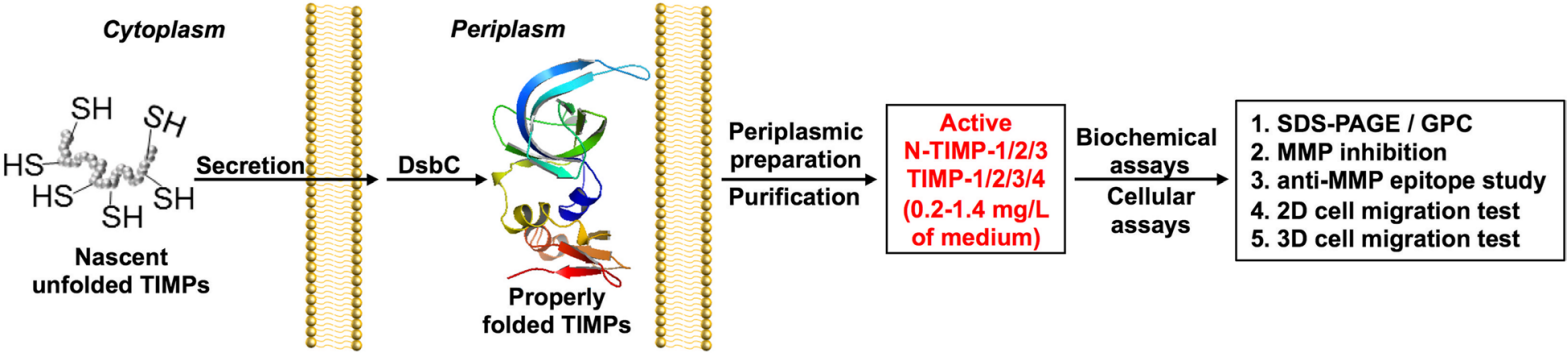
Direct production of soluble (N-)TIMPs in *E. coli* periplasm and their biochemical and cellular function characterizations. Unfolded TIMPs with free cysteines were expressed in cytoplasm and secreted to periplasmic space, where periplasmic chaperones, especially DsbC (a disulfide isomerase), resolved incorrect disulfide bonds, resulting in properly folded TIMPs. Following enzymatic and osmotic treatments, high yields of soluble (N-)TIMPs were purified from periplasmic preparation. The purified (N-)TIMPs were subjected to function tests both biochemically and in the cellular context. *GPC* gel permeation chromatography

Encouraged by functional productions of a variety of recombinant human proteins containing multiple disulfide bonds, i.e. T cell receptors, antibodies and tPA (tissue plasminogen activator) [30–32], in the oxidative periplasm of *E. coli*, we hypothesize that periplasmic expression will result in soluble and active TIMPs. In addition to providing an oxidative environment that promotes the formation of disulfide bonds of (N-)TIMPs, periplasmic expression can enhance the correct protein folding due to multiple molecule chaperons (e.g. SurA, PpiA, Ppid, FkpA, and Skp) [33] and a slow processing rate controlled by secretion machineries [34]. Our previous study directly expressed functional MMP-14 catalytic domain (cdMMP-14) in periplasmic space of *E. coli* [31], and we aim to apply a similar approach for active TIMP production. In this study, by optimizing co-expression of a set of disulfide bond enzymes (Dsb proteins) and selecting a proper expression host, soluble and monomeric (N-)TIMPs were produced in periplasm with high yields (Fig. 1). Periplasmically produced (N-)TIMPs exhibited their biological activities in MMP inhibition assays and cell migration tests. We expect the novel method described here—direct production of functional (N-)TIMPs in *E. coli* without refolding—could greatly expedite many facets of in vitro and in vivo studies associated with metalloproteinases and ECM remodeling.

## Results

### Production of soluble TIMPs in *E. coli* periplasm with high yields

**Fig. 2:**
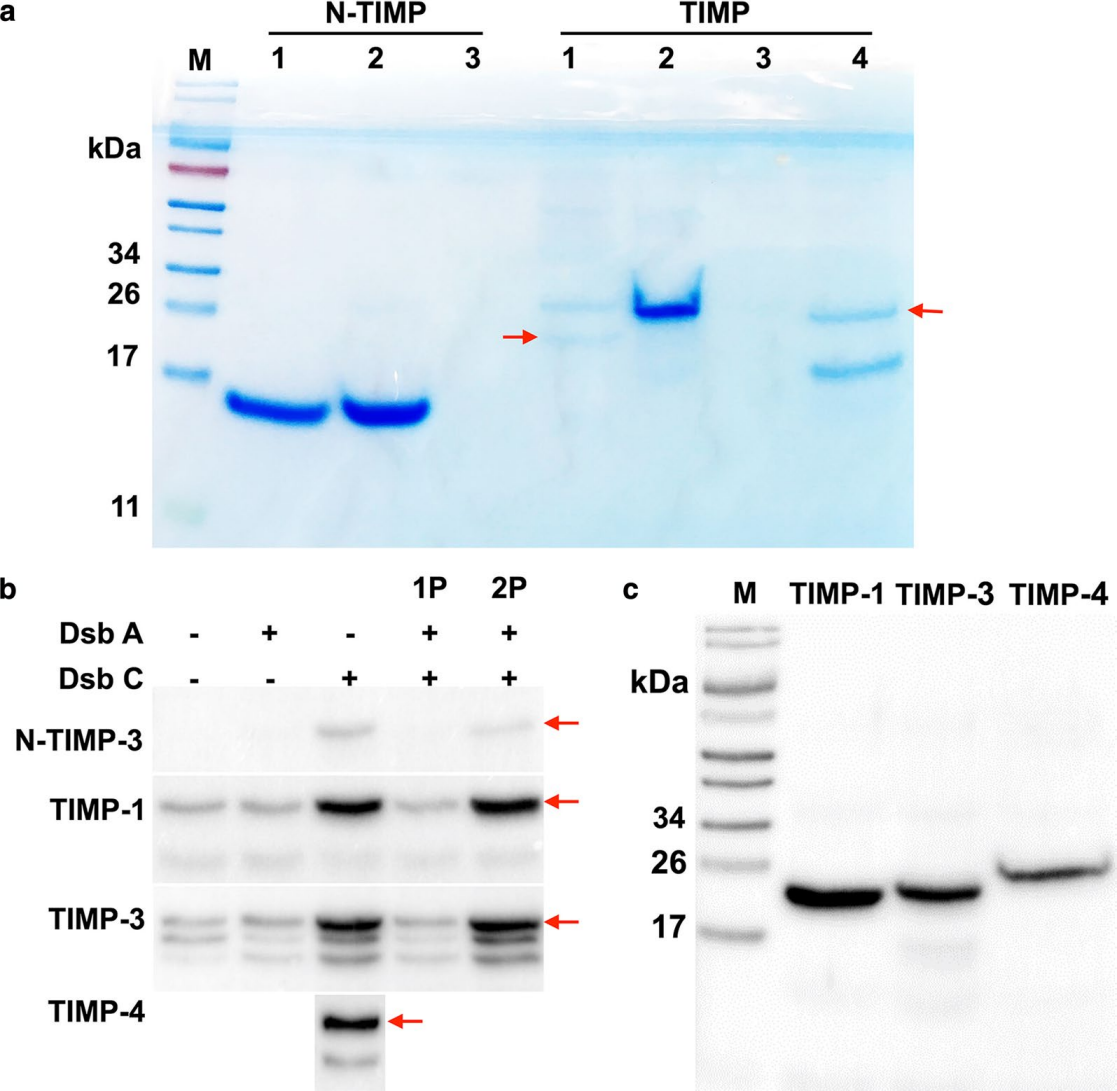
Periplasmic production of (N-)TIMPs and expression condition optimization. **a** Reducing SDS-PAGE of purified (N-)TIMPs stained with Coomassie blue. *Red arrows* indicate the target bands. **b** Effect of periplasmic folding modulators (DsbA and/or DsbC) on expression efficiencies of (N-)TIMPs analyzed by Western blotting using anti-6×His antibody. 1P indicates DsbA and DsbC were under one *P*_*BAD*_ promoter and 2P represents DsbA and DsbC were under two separated *P*_*BAD*_ promoters. *Red arrows* indicate the target bands. **c** Effect of BL21(DE3) on reducing unwanted truncations analyzed by Western blotting. Same amounts of cells were used in **b** and **c**

Full-length TIMP-1/-2/-3/-4 and N-terminal domains of TIMP-1/-2/-3 were constructed at the downstream of a *Plac* promoter and a *pelB* leader peptide sequence. Crystallography of MMP-TIMP complexes suggested that N-terminal residues CXCX of TIMPs directly interact with MMP reaction cleft [35], and TIMP-2 variant with an alanine appended to the amino terminus (Ala+TIMP-2) was inactive [28]. Therefore, a hexa-histidine tag was genetically tagged to the C-termini of (N-)TIMPs for detection and affinity purification. TIMP constructs were transformed to *E. coli* Jude-I for expression. Initial tests indicated that no induction resulted in a higher soluble expression than induction with 1 µM IPTG, a similar phenomenon observed for cdMMP-14 expression [31]. After purification, reducing SDS-PAGE (Fig. 2a) showed single and strong bands of N-TIMP-1/2 (15 kDa) and TIMP-2 (23 kDa), consistent with their calculated MWs. Particularly, 0.5 and 1.4 mg of purified N-TIMP-1/-2 were yielded per liter of culture media. However, TIMP-1/-4 were expressed at much lower levels. Purified TIMP-1 sample showed two bands, one for mature TIMP-1 (22 kDa), and the other band likely associated with unprocessed TIMP-1 having the *pelB* leader signal peptide (27 kDa). In the case of TIMP-4, unwanted truncation was detected at 17 kDa, in addition to the full-length TIMP-4 at 23 kDa, and bands corresponding to N-TIMP-3 and TIMP-3 were not present in their purified samples (Fig. 2a).

Previous studies have demonstrated that periplasmic folding modulators can improve the soluble expression and homogeneity of proteins containing disulfide bonds [32, 36–38]. To increase expression levels of soluble TIMP-1/-3/-4 and N-TIMP-3, we chose DsbA and DsbC as folding modulators to catalyze the formation and isomerization of disulfide bridges, respectively [33]. To investigate the optimal effect of DsbA and/or DsbC, Jude-I cells carrying the TIMP genes were co-transformed with one of four chaperone plasmids: pBAD-DsbA, pBAD-DsbC, pBAD-AC1P (both DsbA and DsbC under one *P*_*BAD*_ promoter), and pBAD-AC2P (both DsbA and DsbC under two separate *P*_*BAD*_ promoters). After cultured in 2×YT media overnight at 30 °C without IPTG, crude periplasmic fractions were prepared for analysis by Western blotting using anti-6×His-HRP. As results shown in Fig. 2b, DsbC (either alone or with DsbA in the 2P format) dramatically raised the yields of TIMP-1/-3 and N-TIMP-3, while DsbA alone did not exhibit significant improvements of TIMP expression. The result that DsbC (disulfide bonds isomerase) was far more efficient than DsbA (a thiol disulfide oxidoreductase) for TIMPs production suggests that presumably resolving the non-native disulfide bonds was the key for proper folding of TIMPs. The suboptimal result of co-expression of both DsbA/C in 1P format was probably due to the inadequate expression level of DsbC that was at downstream of the polycistronic construct. In addition, DsbC alone also significantly enhanced TIMP-4 production at a similar degree as TIMP-1/-3 (Fig. 2b).

Although the amounts of expression were significantly improved by DsbC, unacceptable levels of truncated species were still present in (N-)TIMPs samples (Fig. 2b). We hypothesized that these truncations were digestion products by *E. coli* endogenous proteases, and thus using protease-deficient strain, e.g. BL21 (DE3) could solve the problem. Western blotting analysis confirmed that when BL21 (DE3) was used with DsbC co-expression, truncations were dramatically reduced and only single and strong bands of TIMP-1/-3/-4 were shown (Fig. 2c). With facilitation of DsbC, soluble TIMP-1/-2 were produced in periplasmic fractions of BL21 (DE3) with yields of 0.2 mg purified proteins per liter of 2×YT media.

**Fig. 3:**
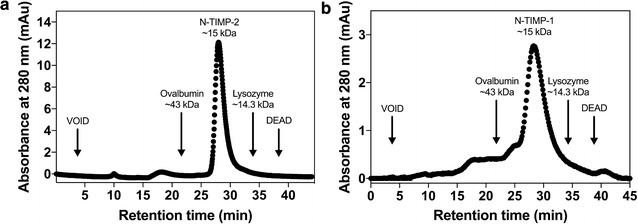
Size exclusion chromatography of purified **a** N-TIMP-2 and **b** N-TIMP-1. The size-exclusion column was equilibrated with 50 mM HEPES (pH 7.5) and 150 mM NaCl. 100 µL of 500 µg/mL N-TIMP-1/-2 was loaded to a superdex™ 75 10/300 GL column (10 mm × 300 mm) at a flow rate of 0.5 mL/min. Chromatograms were obtained by monitoring absorbance at 280 nm. The molecular mass of N-TIMP-1/-2 was estimated by its retention time and comparison with these of standard molecular mass markers, e.g. ovalbumin (43 kDa) and lysozyme (14.3 kDa)

The quality of purified TIMPs was characterized by gel permeation chromatography (GPC). N-TIMP-2 sample displayed a single peak at ~15 kDa associated with its monomer. No aggregates or oligomers were detected, suggesting high quality of the preparation (Fig. 3a). GPC analysis of prepared N-TIMP-1 showed a major peak (~15 kDa) corresponding to its monomer, with presence of trace amounts of oligomers (Fig. 3b). However, compared with N-TIMP-1 sample prepared without DsbC co-expression (Additional file 1: Figure S1), the degree of oligomerization was dramatically reduced, suggesting formation accurate disulfide bonds is critical for TIMPs production.

### Produced TIMPs exhibited expected inhibition potencies towards a panel of MMPs

**Fig. 4:**
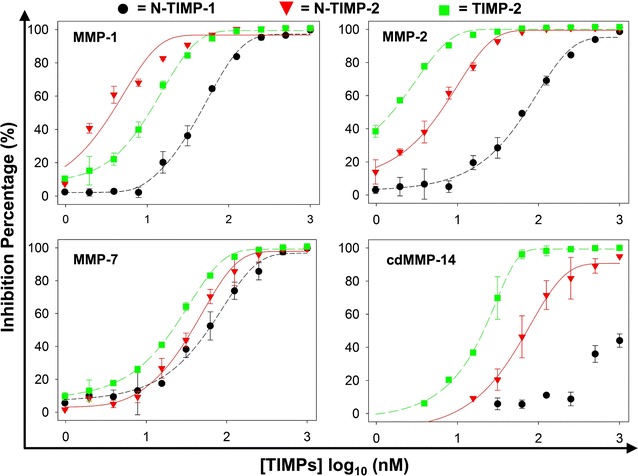
Inhibitory activities of (N-)TIMPs toward MMPs. Semi-quantitative IC_50_s of N-TIMP-1 were determined as 46, 65, and 57 nM for MMP-1/2/7 respectively; IC_50_s of N-TIMP-2 were 3, 6, 38, and 72 nM for MMP-1/2/7 and cdMMP-14 respectively; and IC_50_s of TIMP-2 were 11, 2, 22, and 22 nM for MMP-1/2/7 and cdMMP-14 respectively.* Error bars* represent standard deviations from two independent measurements

Because inhibitions of TIMPs to metalloproteinases are not highly specific, four MMPs were chosen to test the functions of purified (N-)TIMP-1/-2: MMP-1 (collagenase 1), MMP-2 (gelatinase A), MMP-7 (matrilysin), and cdMMP-14 (membrane type 1-MMP). Using quenched fluorescent peptide substrates, MMPs’ enzymatic activities were measured in the presence of 0.98 nM–1 µM purified TIMPs. As results shown in Fig. 4, TIMP-2 and N-TIMP-1/-2 efficiently inhibited MMP-1/-2/-7 cleaving the peptide substrates in a dose-dependent manner with IC_50_ values ranging from 3 to 72 nM. However, N-TIMP-1 was a relatively weak inhibitor to cdMMP-14, an observation consistent with literature [5, 27].

Inhibition constants (*K*_*I*_s) were further calculated for direct comparison with the TIMPs produced through traditional approaches (i.e. mammalian cells or refolding from inclusion body). Results (Table 1) indicated that, except N-TIMP-1 as a weak inhibitor to cdMMP-14, all measured *K*_*I*_s were in nanomolar to subnanomolar scale as expected. Particularly, N-TIMP-1 inhibited MMP-1/-2/-7 with *K*_*I*_s of 7.21 ± 0.33, 10.98 ± 3.76, and 4.62 ± 0.44 nM, respectively. N-TIMP-1 behaves with a similar potency to MMP-7 as reported [27], but its *K*_*I*_s to MMP-1/-2 were 2–27-folds less potent than literature values depending on the studies [21, 24, 27], suggesting the possibility that only portion of prepared N-TIMP-1 was active. However, for periplasmically produced N-TIMP-2, its calculated *K*_*I*_s (0.65 ± 0.14, 0.80 ± 0.03, 2.86 ± 0.94, and 3.96 ± 0.53 nM for MMP-1/-2/-7/-14cd, respectively) well matched with literature data [25, 27], indicating high quality of N-TIMP-2 preparation. The inhibition potencies of TIMP-2 were measured as 2.51 ± 0.01, 0.39 ± 0.09, 2.74 ± 0.09, and 0.34 ± 0.04 nM for MMP-1/-2/-7/-14cd, respectively. Among tested MMPs, only MMP-2 has TIMP-2 inhibition data available in literature, i.e. *K*_*I*_ = 0.6 ± 0.3 nM [28], which well agreed with our results (Table 1). Collectively, both N-TIMP-2 and TIMP-2 exhibited expected *K*_*I*_s, suggesting their correct folding in periplasm. Interestingly, compared with N-TIMP-2, TIMP-2 displayed a significantly higher potency to cdMMP-14, implying that the C-terminal domain of TIMP-2 may contribute recognition of MMPs as well [39].

**Table 1:** Inhibition constants (*K*_*I*_s) of periplasmically produced N-TIMP-1/2 and TIMP-2

TIMPs	*K*_*I*_ (nM) measured in this study/reported in literatures [21, 24, 25, 27, 28]				
	Yield (mg/L)	MMP-1	MMP-2	MMP-7	CdMMP-14
N-TIMP-1	0.5	7.21 ± 0.33/0.4–3	10.98 ± 3.76/0.4–1.11	4.62 ± 0.44/3.6 ± 0.9	Not inhibitory/146
N-TIMP-2	1.4	0.65 ± 0.14/0.4 ± 0.1	0.80 ± 0.03/0.04–0.3	2.86 ± 0.94/2–32	3.96 ± 0.53/0.8–3
TIMP-2	0.2	2.51 ± 0.01/NA	0.39 ± 0.09/0.6 ± 0.3	2.74 ± 0.09/NA	0.34 ± 0.04/NA

All *K*_*I*_ values are exhibited with ±SE

### N-TIMP-2 was functional in antibody epitope characterization

**Fig. 5:**
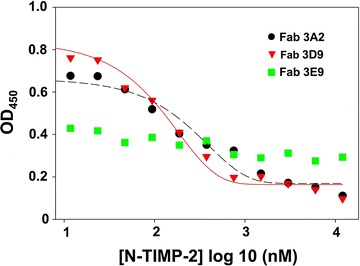
Competitive ELISA between N-TIMP-2 and Fabs for cdMMP-14. Fixed concentrations of Fabs (10 nM for 3A2 and 3D9, and 20 nM for 3E9) were incubated with increasing concentration of N-TIMP-2 on ELISA plates coated with cdMMP-14. The captured Fabs were detected by anti-Fab-HRP for signal development

With structural and inhibition mechanism information available for MMP–TIMP complexes [10, 35, 39], TIMPs have been employed to provide insights on epitopes and affinities of monoclonal antibodies that bind to (and inhibit) MMPs [40]. To investigate whether periplasmically produced TIMPs are capable in such functional assays, competitive ELISAs were conducted in which fixed concentration of Fabs of interest was mixed with increased amounts of TIMP and incubated in wells coated with the associated MMP, and captured Fabs were detected by anti-Fab-HRP for signal development. In our previous studies, a panel of Fabs inhibiting MMP-14 has been isolated from phage display antibody libraries [41]. Three Fabs—3A2 (inhibitory, *K*_*I*_ = 9.4 ± 1.4 nM), 3D9 (inhibitory, *K*_*I*_ = 27 ± 3.5 nM), and 3E9 (non-inhibitory)—were therefore used to test N-TIMP-2 functionality in this study. Results indicated that N-TIMP-2 directly competed with Fabs 3A2 and 3D9 on binding to cdMMP-14 with sigmoidal dose–response curves (Fig. 5). As N-TIMP-2 concentration increased, lower amounts of Fabs 3A2 or 3D9 bound to cdMMP-14. Between these two Fabs, 3A2 responded at a higher N-TIMP-2 concentration, consistent with our measurements that 3A2 is more potent than 3D9 [41]. In contrast, binding of Fab 3E9 to cdMMP-14 did not significantly respond to N-TIMP-2 concentration. These results agreed with our previous observations that Fabs 3A2 and 3D9 bound to MMP-14 reaction cleft vicinity therefore directly competed with N-TIMP-2, while Fab 3E9 was non-inhibitory antibody likely having an epitope not overlapped with the binding site of N-TIMP-2 [41]. Overall, periplasmically produced N-TIMP-2 was successfully used for antibody characterizations by competitive ELISA, an effective method to identify and study antibody inhibitors.

### N-TIMP-1/-2 decreased directionality, and N-TIMP-2 altered migration speed of tumor cells

**Fig. 6:**
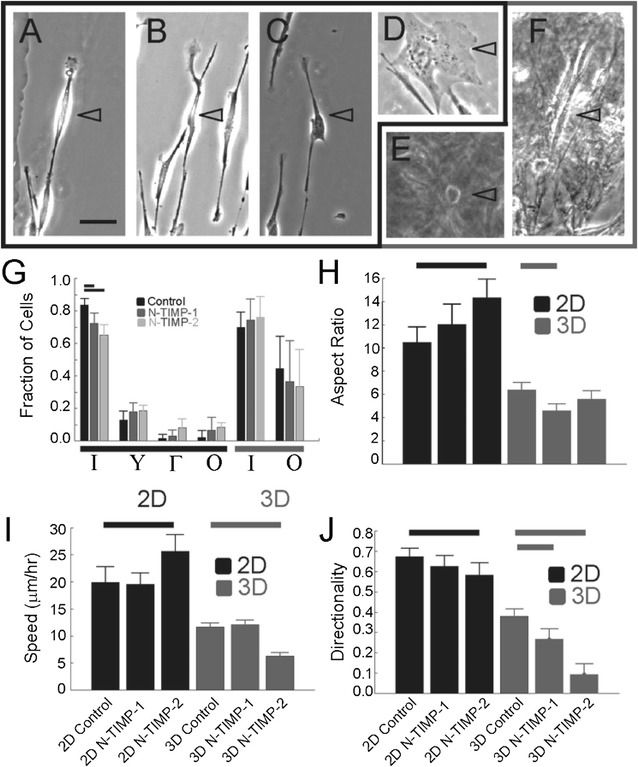
Analysis of N-TIMPs on MDA-MB-231 cell behavior in 2D and 3D contact guidance environments. Cells on 2D substrates (**A**–**D**) or in 3D environments (**E**–**F**) where grouped based on shape and the fraction of cells in each shape were calculated: **A** 2D spindle, **B** 2D branched, **C** 2D bent, **D** 2D spread, **E** 3D spindle and **F** 3D round. *Calibration bar* is 50 μm. **G** Fraction of cells that were of different shapes (*N*_*cells*_ > 79, *N*_*experiments*_ > 3). *Error bars* are 95% confidence intervals calculated using Matlab. **H** Aspect ratio, **I** migration speed and **J** directionality in both 2D (*black*) and 3D (*grey*) environments (*N*_*cells*_ > 79, *N*_*experiments*_ > 3). *Error bars* are 95% confidence intervals calculated using Matlab. Statistical significance was tested using a *t* test with *p* < 0.05 and indicated with* bars*

To test the role of TIMPs in regulating migration, we treated human breast adenocarcinoma MDA-MB-231 cells with 50 nM N-TIMP-1 or -2 and assessed directed cell migration in response to aligned collagen fibers (contact guidance) in 2D and 3D environments. This final concentration was chosen so that MMPs like MMP-2 would be inhibited with either N-TIMP-1 or -2, whereas MMPs like MMP-14 would primarily be inhibited with N-TIMP-2. The cells on 2D contact guidance cues were grouped in four different morphological shapes as shown in Fig. 6A–D. These categories included cells that were spindle shaped (I), branched (Y), bent (Γ) or spread (O). Most cells are identified as spindle shaped, however the addition of N-TIMP-1 or -2 decreased the fraction that were spindle shaped and increased branched, bent and spread, although not significantly. Furthermore, N-TIMP-2 was more effective in forcing this change. The cells suspended in 3D contact guidance cues were grouped in two morphological shapes: spindle shaped (I) or round (O) (Fig. 6E, F). The addition of the N-TIMP-1 or -2 did not have a statistically significant effect on the overall fractions in each category.

While N-TIMP-1 and -2 only marginally affected the cell morphology shape in 2D and 3D contact guidance systems, they significantly affect other attributes. N-TIMP-2 increased the aspect ratio, cell length divided by cell width, during 2D contact guidance, whereas N-TIMP-1 decreases the aspect ratio during 3D contact guidance (Fig. 6H). Cell migration speed responded similarly. While N-TIMP-2 increased speed on 2D contact guidance cues, it decreased speed on 3D contact guidance cues (Fig. 6I). On the other hand, cell directionality changed in the same direction across the 2D and 3D systems. N-TIMP-2 decreases directional fidelity in both 2D and 3D contact guidance environments with the most robust response seen during 3D contact guidance (Fig. 6J). N-TIMP-1, on the other hand, had no effect on directional fidelity in 2D, but decreased directional fidelity in 3D contact guidance system, albeit to a lesser extent than N-TIMP-2.

## Discussion

TIMPs expressed in connective tissues play pivotal roles in extracellular matrix metabolism by inhibiting MMP activities in vivo [2, 3]. Indeed, tumor cell invasion and metastasis can be impeded by up-regulation of TIMPs expression or by an exogenous supply of TIMPs [42–44]. Therefore, a constant and reliable supply of active TIMPs is important for fundamental research and therapeutic development targeting unbalanced metalloproteinases.

Recombinant production of active human TIMPs and their N-terminal domains, usually employs eukaryotic expression systems, chiefly due to disulfide bond formation and glycosylation [6, 7, 9, 21]. Specifically, TIMP-1 has two N-linked glycosylation sites at Asn^30^ and Asn^78^. Although aglycosylated TIMP-1 shows uncompromised inhibition potency, it has been suggested that *N*-glycosylation involves in its folding, solubility and transport to cell surface [6, 7, 45–47]. The glycosylation status of TIMPs has been largely disregarded in clinical samples from patients with cancer, but their role in cancer progression may confound expectations [48]. Glycosylated TIMP-1, but not aglycosylated one, exerts influence of tumor formation and growth in the early phase [49]. Recently, production of N-glycosylated proteins in *E*. *coli* was achieved by utilizing and engineering the N-linked glycosylation system of *Campylobacter jejuni* [50]. With current endeavors to further develop this approach for complicated glycosylations [51], our system would benefit from all these advances for production of fully glycosylated TIMP-1/-3 in *E*. *coli*.

Numerous studies have been conducted to optimize the refolding process, especially to focus on restoring the multiple disulfide bridges [8, 21, 22, 24–29]. Nevertheless, generating biologically active TIMPs from *E. coli* inclusion bodies is considerably challenging, because TIMPs contain 12 cysteine residues, all of which participate in intramolecular disulfide linkages. The aim of present study was to develop an expression system without tiresome refolding process to produce active human TIMPs at high yields that are sufficient for biochemical and cellular experiments. In distinct contrast to previous studies, we employed periplasmic space of *E. coli* to achieve the purpose. By optimizing periplasmic folding modulators in conjunction with expression host and conditions, full length TIMPs and their N-terminal domains were solubly produced at yields of 0.2–1.4 mg purified proteins per liter of culture (Fig. 2). Particularly, periplasmic co-expression of a thiol:disulfide interchange protein DsbC dramatically increased the titers of TIMPs, presumably due to its disulfide isomerization activity by correcting the non-native disulfide bonds, as well as to its general chaperone activity to prevent TIMPs aggregation. Impressively, purified N-TIMP-2 showed as a clean single peak associated with monomer in gel permeation chromatography, without aggregates or oligomeric species presented (Fig. 3a).

The activities and functionalities of periplasmically prepared TIMPs were then confirmed by a set of experiments: (1) inhibition assays against a panel of MMPs, including direct comparison of TIMP-2 and N-TIMP-2 inhibition potencies for the first time (Fig. 4; Table 1); (2) characterization of MMP specific antibodies (Fig. 5); and (3) cell morphology and migration assays in 2D and 3D extracellular matrix (Fig. 6).

To our best knowledge, it is the first study to validate direct production of active human TIMPs in periplasm of *E. coli*. The approach without refolding is straightforward and reliable with less lot-to-lot variation. And high yields and homogeneity of the products indicate that our method is suitable for designed or randomized mutagenesis studies to investigate the biological roles of TIMPs and lead for therapeutic development of engineered TIMP variants. Furthermore, the periplasmic preparation is simple and fast, i.e. the enzymatic and osmotic treatment followed by centrifugation can be finished in ~40 min. In this aspect, we expect that the periplasmic preparation of TIMPs can greatly facilitate downstream processing and make this approach economically attractive.

## Conclusions

Direct production of high yields of active (N-)TIMPs without refolding was achieved in the periplasmic space of protease-deficient *E. coli* strain by co-expressing disulfide isomerase (DsbC). Periplasmically produced TIMPs exhibited expected inhibition potencies towards a panel of MMPs, and functional in competitive ELISA and in a cellular context, i.e. regulating cancer cell morphology and directed migration in 2D and 3D bioassays.

## Methods

### Materials

Restriction enzymes and Vent DNA polymerase were obtained from NEB. Human MMPs-1/2/7 were purchased from AnaSpec. The catalytic domain of human MMP-14 (cdMMP-14) was produced and biotinylated as previously described [31]. The following vectors and cell lines were from lab stock: periplasmic expression vector pMopac16 [52], pBAD33 [53], DsbA/C chaperon plasmids [54] pBAD-DsbA, pBAD-DsbC, pBAD-AC1P (both DsbA and DsbC under one *P*_*BAD*_ promoter) and pBAD-AC2P (DsbA and DsbC under two separated *P*_*BAD*_ promoters), and *E. coli* strains Jude-I [DH10B F’::Tn10(Tet^r^)] and BL21(DE3). A human mammary basal/claudin low carcinoma cell line (MDA-MB- 231, ATCC) was cultured in Dulbecco’s Modified Eagles Medium (DMEM) (Sigma-Aldrich) containing 10% fetal bovine serum (FBS) (Gibco) and 1% penicillin–streptomycin (pen-strep) (Gibco) at 37 °C in 5% CO_2_. Imaging media for MDA-MB-231 cells was the same as the subculturing media, with the exception that no phenol red was included and that 12 mM HEPES (Sigma-Aldrich) was included.

### Cloning and expression of (N-)TIMPs

The DNA fragments encoding human TIMP-1/2/3/4 were synthesized (IDT) and cloned into pMopac16, which carries a *lac* promoter and a *pelB* leader. The genes of N-TIMP-1/-2/-3 were PCR amplified (residues 1–126, 1–127 and 1–121 respectively) and cloned into pMopac16. Transformed *E. coli* Jude-I cells were grown in 2×YT media supplemented with 35 µg/mL chloramphenicol (2×YT/Chlor) for (N-)TIMPs expression overnight at 30 °C without IPTG. For co-expression with periplasmic folding modulators, the chloramphenicol acetyltransferase genes on the chaperone plasmids were replaced with β-lactamase genes. Double transformed Jude-I or BL21(DE3) containing pBAD-DsbA/-DsbC/-AC1P/-AC2P and pMopac16-(N-)TIMPs were cultured at 30 °C overnight in 2×YT media supplemented with 100 µg/mL ampicillin, 35 µg/mL chloramphenicol and 0.2% arabinose (2×YT/Amp/Chlor/arabinose) without IPTG.

### Periplasmic fractions preparation and recombinant (N-)TIMPs purification

Periplasmic fractions were prepared as previously reported [31]. Briefly, three OD cells were harvested and resuspended in 100 μL 200 mM Tris–HCl pH 7.5, 20% sucrose, 30 U/μL lysozyme, and then incubated for 10 min at room temperature. The mixture was then treated by osmotic shock with 100 μL ice-cold ddH_2_O followed by incubation on ice for 10 min. The mixture was centrifuged at 15,000×*g* for 2 min, and the recovered supernatant was analyzed by SDS-PAGE and Western blotting with anti-6×His-HRP (Abcam) and chemiluminescent substrate (Thermo Scientific). The (N-)TIMPs were purified from periplasmic preparations by Ni–NTA agarose beads following manufacturer’s protocols (Qiagen). The homogeneity of purified proteins was confirmed by SDS-PAGE. Overnight dialysis was conducted to remove residual imidazole, and the concentrations of purified (N-)TIMPs were determined by NanoDrop (Thermo), before storage in 20% glycerol at −80 °C.

### Gel permeation chromatography

GPC analyses were performed on an ÄKTAprime using a superdex 75 10/300 GL size-exclusion column (GE Healthcare) equilibrated with 50 mM HEPES pH 7.5, 150 mM NaCl. 100 µL 500 µg/mL N-TIMP-2 was injected at a flow rate of 0.5 mL/min to obtain chromatograms of absorbance at 280 nm. The molecular mass of N-TIMP-2 was estimated based on the retention times of standard proteins including ovalbumin (43 kDa) and lysozyme (14.3 kDa) (Sigma-Aldrich).

### FRET inhibition assay

Concentrations of active MMPs were titrated by GM6001 (a highly potent broad-spectrum MMP inhibitor, EMD). In 96-well black assay plates (Corning), 60/60/250/500 nM MMP-1/-2/-7/-14cd was incubated with 0.98–1000 nM (N-)TIMPs for 30 min at room temperature, and 1 μM M-2350 substrate (Bachem) was added to start the reactions. The fluorescent signals with excitation at 328 nm and emission at 393 nm were monitored using a Synergy H4 microplate reader (BioTek). Initial velocities were calculated to determine IC_50_, and inhibition constants (*K*_*I*_s) were obtained using the following equation for slow tight-binding inhibitors [5]:

1 $$\frac{{[I]_{t} }}{{1 - \frac{{v_{s} }}{{v_{0} }}}} = K_{I} \frac{{v_{o} }}{{v_{s} }} + [E]_{t}$$

[*I*]_*t*_ is the total inhibitor concentration, [*E*]_*t*_ is the total enzyme concentration. *v*_0_ is the rate of substrate hydrolysis in the absence of TIMP (M · s^−1^), *v*_*s*_ is the equilibrium rate of substrate hydrolysis reached after inhibition (rate of hydrolysis in the inhibited steady state) (M · s^−1^) for each TIMP concentration, [*I*]_*t*_.

### Competitive ELISA

12 μM N-TIMP-2 was two-fold serially diluted and incubated with Fabs of interest (10 nM 3A2, 10 nM 3D9, or 20 nM 3E9) for 15 min in streptavidin microplate wells coated with 5 μg/mL biotinylated cdMMP-14. After washing, bound Fabs were detected by anti-Fab-HRP and TMB solution, then stopped with 1 M H_2_SO_4_. OD_450_ signals were measured using a microplate reader.

### Imaging at 2D environment

For 2D cell imaging, a 15 × 15 mm piece of muscovite mica (highest grade VI, Ted Pella, Redding, CA, USA) was freshly cleaved using tape. High concentration non-pepsin treated rat tail collagen type I (Corning, Corning, NY, USA) was diluted to 10 μg/mL in 50 mM Tris–HCl pH 9.2 and 200 mM KCl, and incubated on the mica at room temperature for 3–6 h [55]. After incubation, the mica was washed with distilled water, laid against the edge of a tissue culture dish then allowed to dry overnight. Collagen substrates were seeded with MDA-MB-231 cells (~6000 cells/cm^2^), which were treated with 50 nM N-TIMP-1 or N-TIMP-2. After 2 h of incubation, substrates with cells attached to the fibrils were inverted onto two strips of double-sided tape attached to a microscope slide to generate a flow chamber. The chamber was filled with imaging media, sealed and imaged by phase contrast microscopy on a heated stage at 37 °C every 2 min for 12 h.

### Cell imaging in 3D gels

For 3D imaging, the cells were trypsinized and suspended at a concentration of 600,000 cells/mL in a 2 mg/mL non-pepsin-treated rat collagen type I solution. 50 nM N-TIMP-1 and N-TIMP-2 were added to this solution prior to the addition of the collagen. A 125 μL volume of the solution was pipetted into a MatTek dish (MatTek Corporation, Ashland, MA, USA) and allowed to polymerize at room temperature for 45 min. After polymerization, 125 μL of imaging media was added to the top of the gel. To seal the chamber, a 5-mm thick pad of polydimethylsiloxane (PDMS) (Dow) punctured with an acupuncture needle was placed on top and sealed with food grade lubricant (McGlaughlin Oil Company, Columbus, OH). The acupuncture needle was then rotated one full rotation to align the collagen fibers and placed in a 37 °C incubator. After 24 h, the chambers were removed from the incubator and imaged every 2 min for 8 h. The images were analyzed using the MTrackJ plugin in ImageJ which allows the user to track the cells on an image-by-image basis. The plugin generates the *x*–*y* coordinates of the cell at each time point. Trajectories were analyzed using a custom Matlab script to calculate a migration speed. Directionality was determined using another custom Matlab script. The angle between the direction of the migration and a radial vector field originating from the needle was used to calculate directionality with the following equation:

2 $$DI = \cos 2\theta$$

Aspect ratio was calculated by measuring the length of a cell and dividing it by the width of that same cell. Analysis of cell morphology was completed by binning cell shape based on the shape created by the extensions after the cells had time to adhere to its environment: 9 h for 2D and 24 h for 3D. 95% confidence intervals were calculated using *Matlab* with a sample of at least 79 cells. A two tailed *t* test at *p* ≥ 0.05 was conducted to identify statistical differences between the N-TIMP conditions and that of the control.

## Additional file

**Additional file 1. Figure S1.** Size exclusion chromatography of purified N-TIMP-1 prepared without DsbC co-expression. The size-exclusion column was equilibrated with 50 mM HEPES (pH 7.5) and 150 mM NaCl. 100 µL of 500 µg/mL N-TIMP-1 was loaded to a superdex™ 75 10/300 GL column (10 mm × 300 mm) at a flow rate of 0.5 mL/min. Chromatograms were obtained by monitoring absorbance at 280 nm. The molecular mass of N-TIMP-1 was estimated by its retention time and comparison with these of standard molecular mass markers, e.g. ovalbumin (43 kDa) and lysozyme (14.3 kDa).

## Abbreviations

TIMP: tissue inhibitor of metalloproteinase
MMP: matrix metalloproteinase
ECM: extracellular matrix
ADAM: a disintegrin and metalloproteinase
ADAMTS: a disintegrin and metalloproteinase with thrombospondin motifs
DsbA and DsbC: disulfide oxidoreductases A and C
