# Supplementary material for: Direct expression of active human tissue inhibitors of metalloproteinases by periplasmic secretion in Escherichia coli
Source: Microb Cell Fact. 2017 Apr 28;16:73. doi: 10.1186/s12934-017-0686-9 (PMC5410052; doi:10.1186/s12934-017-0686-9)
Supplement: Supplementary file 1 — Additional file 1. Figure S1. Size exclusion chromatography of purified N-TIMP-1 prepared without DsbC co-expression. The size-exclusion column was equilibrated with 50 mM HEPES (pH 7.5) and 150 mM NaCl. 100 µL of 500 µg/mL N-TIMP-1 was loaded to a superdex™ 75 10/300 GL column (10 mm × 300 mm) at a flow rate of 0.5 mL/min. Chromatograms were obtained by monitoring absorbance at 280 nm. The molecular mass of N-TIMP-1 was estimated by its retention time and comparison with these of standard molecular mass markers, e.g. ovalbumin (43 kDa) and lysozyme (14.3 kDa). [file 12934_2017_686_MOESM1_ESM.docx]

**Supplementary Data**

Direct expression of active human tissue inhibitors of metalloproteinases by periplasmic secretion in *Escherichia coli*

Ki Baek Lee^1^, Dong Hyun Nam^1^, Jacob A Nuhn^2^, Juan Wang^2^, Ian C Schneider^2^, Xin Ge^1, *^

^1^Department of Chemical and Environmental Engineering, University of California, Riverside, 900 University Ave, Riverside, CA 92521; ^2^Department of Chemical and Biological Engineering, Iowa State University, 3053 Sweeney, Ames, IA 50011

**
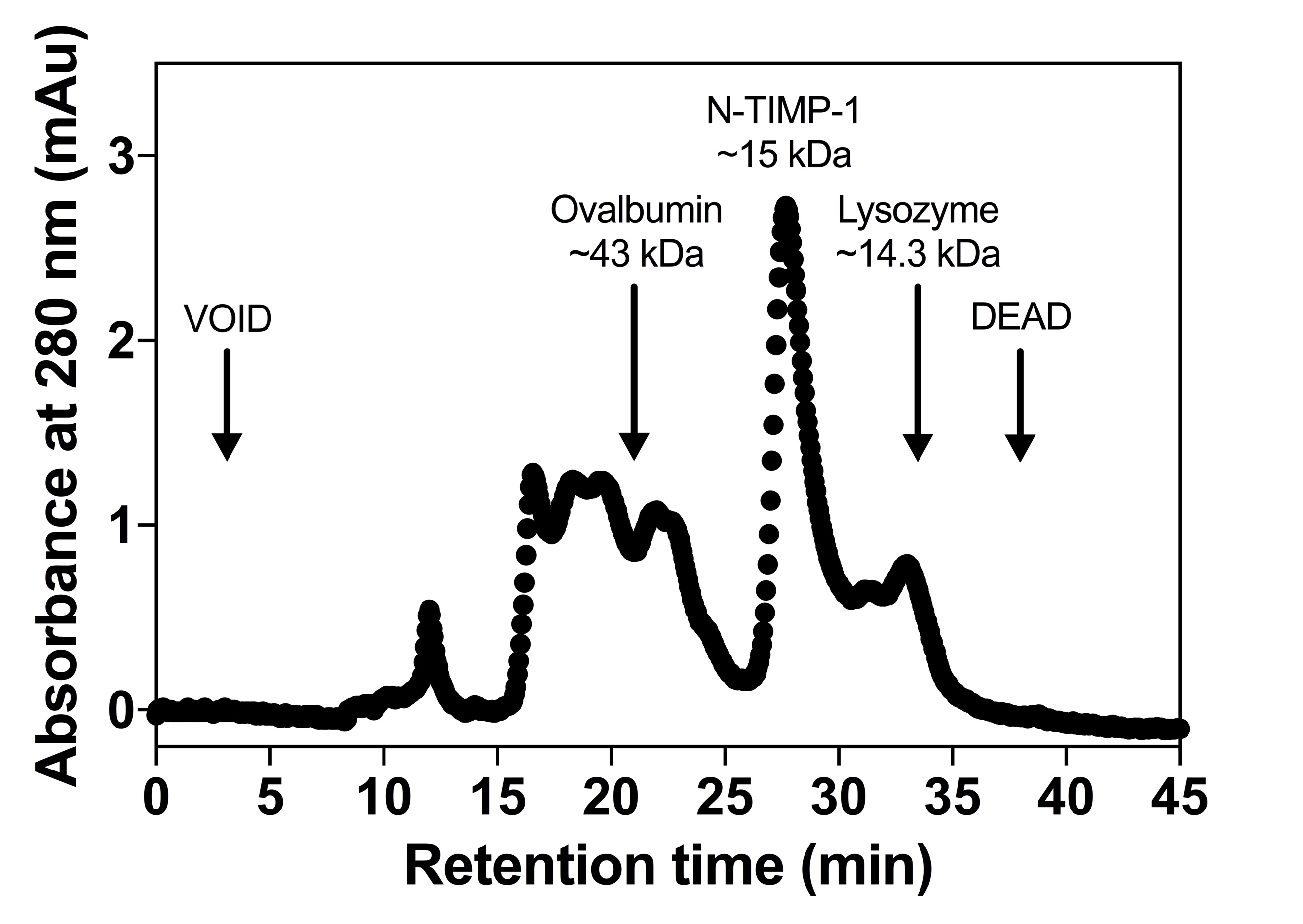
**

**Supplementary Figure 1. Size exclusion chromatography of purified N-TIMP-1 prepared without DsbC co-expression.** The size-exclusion column was equilibrated with 50 mM HEPES (pH 7.5) and 150 mM NaCl. 100 µL of 500 µg/mL N-TIMP-1 was loaded to a superdex^TM^ 75 10/300 GL column (10 mm × 300 mm) at a flow rate of 0.5 mL/min. Chromatograms were obtained by monitoring absorbance at 280 nm. The molecular mass of N-TIMP-1 was estimated by its retention time and comparison with these of standard molecular mass markers, e.g. ovalbumin (43 kDa) and lysozyme (14.3 kDa).
